# Protein design accelerates the development and application of optogenetic tools

**DOI:** 10.1016/j.csbj.2025.02.014

**Published:** 2025-02-21

**Authors:** Lingyun Zhu, Yuxuan Wang, Xiaomin Wu, Guohua Wu, Guohao Zhang, Chuanyang Liu, Shaowei Zhang

**Affiliations:** Department of Biology and Chemistry, College of Sciences, National University of Defense Technology, Changsha, Hunan 410073, China

**Keywords:** Protein design, Optogenetics, Photosensitive domains, Machine learning

## Abstract

Optogenetics has substantially enhanced our understanding of biological processes by enabling high-precision tracking and manipulation of individual cells. It relies on photosensitive proteins to monitor and control cellular activities, thereby paving the way for significant advancements in complex system research. Photosensitive proteins play a vital role in the development of optogenetics, facilitating the establishment of cutting-edge methods. Recent breakthroughs in protein design have opened up opportunities to develop protein-based tools that can precisely manipulate and monitor cellular activities. These advancements will significantly accelerate the development and application of optogenetic tools. This article emphasizes the pivotal role of protein design in the development of optogenetic tools, offering insights into potential future directions. We begin by providing an introduction to the historical development and fundamental principles of optogenetics, followed by an exploration of the operational mechanisms of key photosensitive domains, which includes clarifying the conformational changes they undergo in response to light, such as allosteric modulation and dimerization processes. Building on this foundation, we reveal the development of protein design tools that will enable the creation of even more sophisticated optogenetic techniques.

## Graphical Abstract

**Figure:**
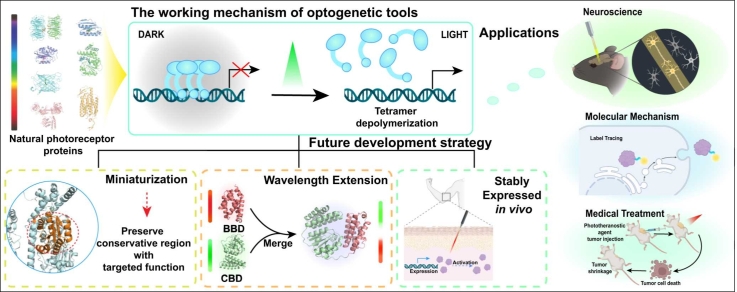

## 1 The emergence of optogenetics

Traditional cell biology research has long been hinded by technological limitations in controlling and manipulating cellular processes. Conventional biological techniques, such as electrophysiology, pharmacological interventions, and gene knockout approaches, offer critical insights but are plagued by limited precision in modulating specific cell populations or dynamic processes. These methods often exhibit **lower spatiotemporal resolution**, thus restricting precise control over the continuous dynamic processes within cells. Additionally, they typically **fall short of achieving the independent modulation of specific cell populations or individual cell types**. Furthermore, certain intervention techniques may **provoke non-specific reactions** in cells or organisms, consequently resulting in imprecise experimental outcomes.

By fusing fluorescent proteins with target proteins, researchers accomplished the visualization of specific molecules and processes within cells [1], [2]. Seminal work by Chalfie *et al.*[3] and Inouye *et al.*[4] established green fluorescent protein (GFP) as a universal marker for tracking gene expression dynamics, laying the groundwork for integrating light-based tools in cellular studies. While fluorescent proteins like GFP revolutionized cellular imaging, their role in optogenetics is distinct from photoactuators that enable direct cellular manipulation. However, in the pursuit of high-resolution control over cellular functions, scientists have turned to naturally occurring light-responsive organisms. Light, as a form of energy, is easily modulated and highly directional, offering great potential for precise control in both spatial and temporal dimensions. The ability of plants and other photosensitive organisms to respond to light signals, in processes like photosynthesis and visual perception, has inspired researchers to explore the use of light as a tool for manipulating cellular functions [5], [6]. Light perception in biological systems is mediated by photoreceptive elements, which range from specialized cells (e.g., retinal light-detecting cells in animals) to molecular photoactuators (e.g., microbial opsins or plant phytochromes). These components convert light energy into biochemical or electrical signals, termed photosensitive proteins. Within photosensitive proteins, the photosensitive domain absorbs specific wavelengths of light, which triggers conformational changes that initiate a cascade of signal responses. These wavelength-specific conformational changes subsequently activate cellular signaling pathways, ultimately regulating cellular physiology. Most photosensitive proteins are found in plants, where they play crucial roles in mediating cellular responses to light, including phytochromes, cryptochromes, phototropins, and UV Resistance Locus 8 (UVR8) [7], [8]. Photosensitive proteins are also present in animals and bacteria, such as opsins, blue light using flavin (BLUF) proteins and light-oxygen-voltage sensing domain (LOV) proteins, which participate in both visual and non-visual physiological functions, responsible for receiving light signals and transducing them into cellular signals [9].

The spatiotemporal specificity and sensitivity of photoreceptive components in plants and other organisms are crucial for advancements in cell biology and synthetic biology. In 2002, photosensitive proteins were first introduced into cellular biology research, with notable contributions from Shimizu-Sato, who established a promoter system to regulate gene expression [10], and Zemelman, who developed an optogenetic approach to stimulate neuronal populations [11]. Since then, photosensitive proteins have progressed rapidly in conjunction with neuroscience. In 2005, Boyden *et al.* developed a technique that could alter neural processing at the level of single spikes and synaptic events using light, pioneering research on Channelrhodopsin-2(ChR2), achieving the precise activation of neuronal activity in mammalian neurons,a rapidly gated light-sensitive cation channel [12], [13]. The fundamental discovery in a non-mammalian system laid the groundwork for subsequent optogenetic applications in mammalian neurons. Notably, microbial opsins such as ChR2 and halorhodopsin(NpHR) from *Natronomonas pharaonic* were instrumental in establishing optogenetics as a field, demonstrating that light-sensitive ion channels from simple organisms could be utilized to control neuronal activity with millisecond-precision [14]. These early efforts and investigations in cellular biology laid the groundwork for comprehending and leveraging the potential of light-based control mechanisms.

In 2006, Deisseroth *et al.* coined the term "optogenetics", emphasizing reversible, cell-type-specific modulation via genetically encoded light sensors [15]. Following its introduction, research on optogenetics has expanded rapidly, with the number of publications increasing exponentially at an average annual growth rate doubling approximately every two years.

**Fig. 1:**
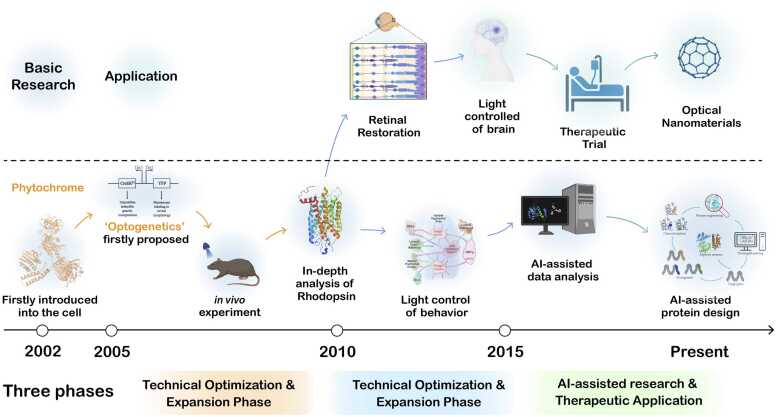
Development of optogenetics from emergence to present. The diagram shows the development process of optogenetics from aspects of basic research and application development. The key events in the process are highlighted in the figure.

The development of optogenetics can be classified into three main stages (Fig. 1). The **Initial Development Phase (2005–2010)**: During this stage, optogenetics was characterized by the introduction of light-sensitive proteins derived from microbial sources. Fundamental work laid the principles and techniques of optogenetics, primarily employed for controlling neuronal activity and developing therapeutic strategies [13], [16] for neurological disorders. There was growing recognition of optogenetics' potential to target a broader range of biological systems beyond neuroscience [17].

The second phase, **Optimization and Expansion Phase (2010–2015)**: This period was marked by the continuous optimization of optogenetic techniques and the expansion of their applications. Significant technological advancements included the development of novel optogenetic actuators [18] and delivery methods [19], as well as the integration of optogenetics with other technologies [20], [21]. Optogenetics began to pervade through various research areas, including mood disorders, addiction, epilepsy, cardiac physiology, and neural circuit analysis [22], [23], [24], [25].

The third phase is the **AI-assisted Research and Therapeutic Application (2015–Present)**: In this latest phase, optogenetics has achieved key breakthroughs in several fields, particularly in the treatment of neurological diseases [26], regenerative medicine [27], [28]. Research experiments in animal models have marked the gradual transition of optogenetics to clinical applications. Notably, AI-assisted protein designs have emerged as a transformative approach, enabling the rapid discovery and optimization of optogenetic tools. For example, AlphaFold predicted microbial opsin structures, accelerating discovery of improved variants [29], RFdiffusion designed *de novo* light-sensitive proteins like red-shifted ChR2 for deeper tissue applications [30]. Recent computational approaches have also facilitated the development of bistable optogenetic tools such as the inhibitory optoGPCR, which enables multiplexed optical control of neural circuits with high temporal precision and minimal cross-talk [31]. Recent innovations in optogenetics involve the development of new photosensitive proteins through protein engineering and computational protein design to meet the diverse control requirements of different biological functions.

Since its formal introduction in 2005, optogenetics has undergone substantial development across these three stages. Initial research focused on neuronal activity and its implications for neurological diseases. Subsequent advances in actuators and delivery methods expanded optogenetic applications to cancer, epilepsy, and other fields. Beyond neuroscience, optogenetics has also brought about major changes in synthetic biology. For instance, Toettcher *et al.* reported a system based on light feedback for precisely controlling the dynamic process of intracellular signal transduction [32]. In recent years, the continuous refinement of optogenetic control over biological activities has advanced medical fields related to the nervous system and regenerative medicine. Concurrently, computational protein design has enabled the development of new optogenetic tools, further broadening the scope and efficacy of optogenetic applications.

## 2 Photosensitive domains

**Fig. 2:**
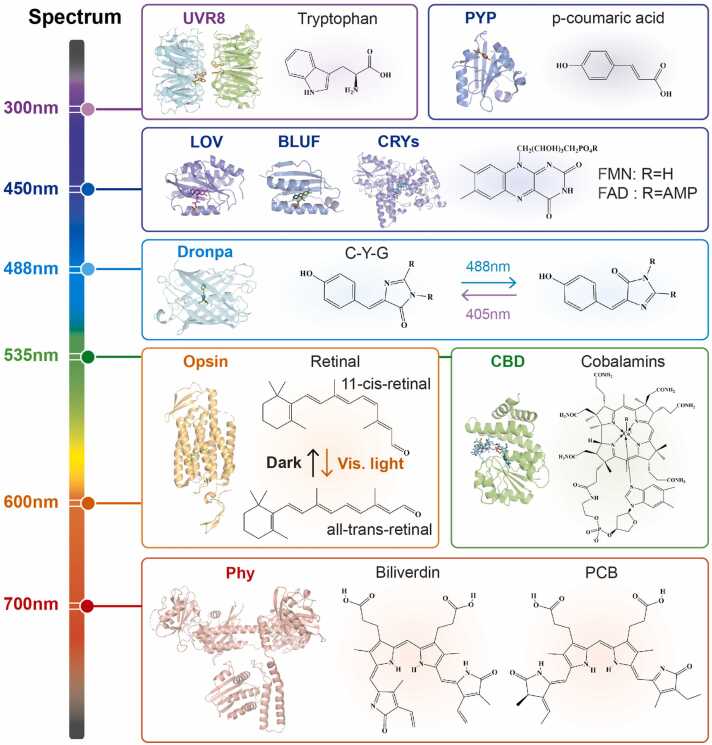
Schematic of the photosensitive domains responding to various wavelengths. The different photosensitive domains and their light sensing chromphores are illustrated in the figure, along with the corresponding regions of the visible spectrum in which they respond. Abbreviations: UVR8, UV resistance 8; PYP, Photoactive Yellow Protein; LOV, light-oxygen-voltage-sensing; BLUF, Blue-Light-Using Flavin Adenine Dinucleotide-Containing; CRY, cryptochrome; FAD, flavin adenine; FMN, flavin mononucleotide; C-Y-G, cysteine-tyrosine-glycine; CBD, cobalamin-binding domain; Phy, Phytochrome; PCB, phycocyanobilin.

**Fig. 3:**
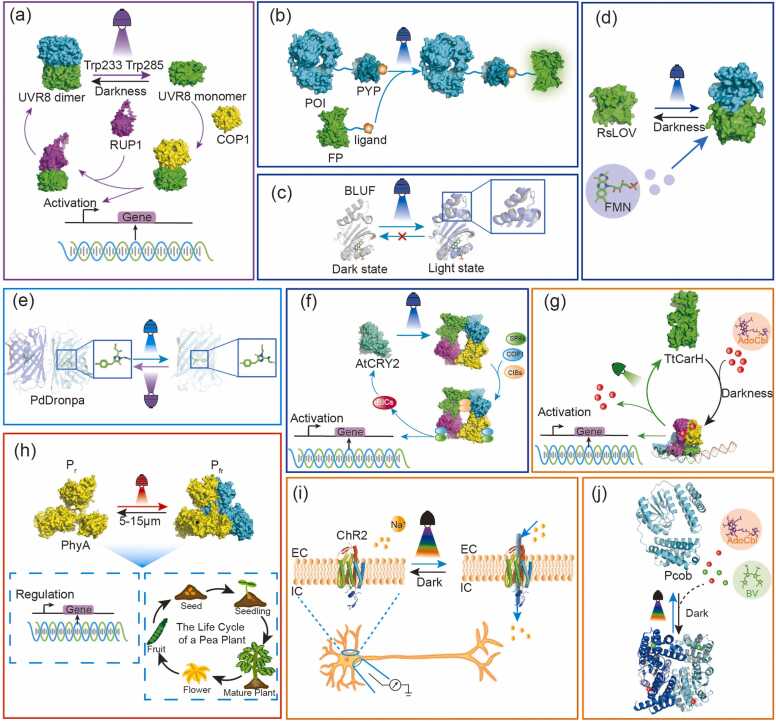
Schematic diagram of the regulatory processes of different photosensitive domains under specific light conditions. Working mechanism of photosensitive domains. The color of the light refers to the wavelength of the response, and the protein polymers are colored by monomer chains. Abbreviations: COP1, Constitutively Photomorphogenic 1; RUP1, Repressor of UV-B Photomorphogenesis 1; Pr, phytochrome red; Pfr, phytochrome far-red; BIC, blue-light inhibitor of cryptochrome; SPA, suppressor of phytochrome A; CIB, cryptochrome-interacting basic helix-loop-helix; EC, extracellular component; IC, intracellular component; Pcob, Photocobilin.

Optogenetic tools have been modularized through the integrating protein of interest with foundational photosensitive proteins [33]. Among the applications of optogenetics, the pivotal element is a highly effective photosensitive protein that has evolved from plants and microorganisms to transduce environmental light into intracellular signals regulating their life cycle. This protein typically comprises a photosensitive domain and an effector module in general [34], [35]. The photosensitive domains convert photo energy into biochemical signals via light-triggered conformational changes [36]. Most rapidly revert to their ground state upon light withdrawal, enabling their use as optical molecular switches. The evolutionary development of photosensitive domains reflects organisms' adaptation to harness light energy. Building on these natural templates, synthetic photosensitive domains have been engineered to enhance optogenetic control. This section systematically reviews key photosensory domains, focusing on their spectral characteristics, cofactor requirements, and photoresponse mechanisms (Fig. 2, Fig. 3).

### 2.1 UVR8

The UVR8 photosensitive domain derived from the *Arabidopsis* gene UVR8, is unique in its ability to absorb UV-B radiation (280–315 nm) [37]. Distinct from conventional photosensitive domains, UVR8 utilizes intrinsic tryptophan residues (Trp285 and Trp233) to detect UV-B instead of the customary chromophores [38]. Upon UV-B absorption, UVR8 transitions from a homodimeric (inactive) state to a monomeric state, interacting with E3 ubiquitin ligase COP1, thereby initiating UV-B signal transduction and altering gene expression [39] (Fig. 3a).

### 2.2 Photoactive yellow protein

Photoactive Yellow Protein (PYP), a 14-kDa photosensitive domain responsible for negative phototaxis in *Halorhodospira halophila*, encapsulates a p-hydroxycinnamic acid chromophore linked to the amino acid residue Cys69 [40]. The photosensitive effect of PYP is a completely self-contained photocycle involving a trans-to-cis isomerization of the chromophore, activated by blue light (peak wavelength is 450 nm), with microsecond-scale intermediate states [41], [42] (Fig. 3b).

### 2.3 LOV

LOV, a subgroup of the Per-ARNT-Sim superfamily, was initially identified in photoresponsive genes in plants [43]. They bind to flavin mononucleotide (FMN) and exhibit a peak absorption at 447 nm in the absence of light, designated LOV_447_[44]. Upon light exposure, a covalent bond forms between the chromophore's C(4a) atom and a conserved cysteine within the active site, producing an adduct (LOV_390_) with peak absorbance at 390 nm [45] (Fig. 3d). Engineered LOV domains have been widely applied for spatiotemporal gene regulation. For example, the LOV-LexA system enables precise light-gated transcriptional control by coupling LOV conformational changes to DNA-binding domains [46]. Similarly, PhotoGal4 in Drosophila explants exemplifies the versatility of LOV-based switches for tissue-specific optogenetic manipulation [47], further demonstrating the modularity of photosensitive domains in synthetic biology applications.

### 2.4 BLUF

BLUF domain comprises a series of photosensitive domains that sense blue light through flavin adenine dinucleotide (FAD), first discovered in AppA of *Rbodobacter spbaeroides*, mainly existing in prokaryotes [48]. Unlike LOV domains, BLUF domains exhibit minimal FAD structural changes upon illumination (Fig. 3c). In the dark and light states, the absorption spectrum of the FAD flavin loop is redshifted by 10 nm within 1 ns, and the recovery of the ground state is completed within 30 minutes.

### 2.5 Cryptochromes

Cryptochromes (CRYs) are flavoproteins regulating plant photomorphogenesis [49] and animal circadian rhythms [50], [51]. CRY proteins are categorized into three groups: CRY1 and CRY2 are found in Arabidopsis, while CRY3 (CRY-DASH) is present across various species such as Drosophila and humans [52], [53]. Among them, CRY2 is the most widely studied cryptochrome. Upon exposure to blue light, the FAD bound to a conserved amino-terminal photolyase-related domain (PHR) undergoes a conformational change, releasing the variable-length carboxy-terminal domain (CCT). This allows PHR and CTT to cooperate as a homodimer, whereas CRY2 remains in its monomeric form under dark conditions [48], [53], [54], [55] (Fig. 3f).

### 2.6 Opsins

Opsins are G-protein-coupled receptors characterized by a seven-transmembrane architecture and differentiated into several subfamilies via molecular phylogenetic analysis. A distinguishing feature of opsins is a lysine residue (K296) on the seventh helix, functioning as the retinal-binding site. When the visual chromophore, 11-cis-retinal, attached to K296, it undergoes photoisomerization to all-trans-retinal across the ultraviolet to orange light spectrum [56]. The photoreceptive properties of opsins, including variants from microorganisms such as halorhodopsin and channelrhodopsin (ChR), have enabled their application in the precise control of neuronal activity [57], [58] (Fig. 3i). Notably, Janovjak *et al.* engineered light-activated ion channels with enhanced temporal resolution, providing a framework for remote control of neuronal firing dynamics *in vivo*[59]. Among them, the resolved crystal structure of Chrimson, a red light-activated ChR, revealed key residues governing its spectral tuning and kinetics [60]. The finding directly inform the design of variants with enhanced tissue penetration and temporal precision.

### 2.7 Dronpa

Dronpa, a photoswitchable fluorescent protein derived from a Pectiniidae coral GFP variant, was first engineered in 2004 by Ando *et al.* to enable reversible single-molecular photoswitching. It can convert to a dim state at 488 nm and revert to a bright state at 405 nm with a rapid response time measured in milliseconds [61] (Fig. 3e). The on-state, characterized by a cis-conformation, is achieved through a cis-trans isomerization triggered by the exposure of light at 488 nm. This process involves a position change in four residues (Arg66, Ser142, Val157, His193), which are located adjacent to the chromophore cavity. Notably, this structual modification does not influence the protonation equilibria[62], which is a testament to the specificity and precision of its molecular design, allowing for controlled fluorescence modulation without compromising the integrity of the chromophore's chemical properties.

### 2.8 Cobalamin-binding domain

Cobalamin is an essential component that plays a crucial role in human and animals as a cofactor for many enzymes. The central feature of cobalamin is the corrin ring, which binds to various proteins to facilitate enzymatic activity. Notably, many proteins without identified enzymatic function also contain a cobalamin-binding domain [63], [64], highlighting the complex interactions between cobalamin and protein structures. Researchers have found that cobalamin-binding domain (CBD) in CarH responds to light across different wavelengths, including UV (280–400 nm), blue (400–500 nm), green (500–570 nm). This photoreactivity enables the manipulation of transcription mediated by 5′-deoxyadenosylcobalamin (AdoB_12_), one of the *in-vivo* forms of vitamin B_12_ [65]. In the dark, CarH forms a tetramer consisting of two head-to-tail dimers upon binding to AdoB_12_. This tetrameric complex then binds to operator DNA, upon illumination, the tetramer dissociates, allowing transcription to resume [66], [67] (Fig. 3g). The new biological role of B_12_ and the light sensing function of CBD lead to new possibilities for component design in optogenetics and also fill the vacancy of green-light-responsive tools [67].

### 2.9 Phytochromes

Phytochromes are plant-origin photosensitive domains that exploit the dynamic light environment for optimal growth by switching between a red-absorbing state (P_r_, up to 660 nm) and a far-red-absorbing state (P_fr_, up to 700 nm). This switch is mediated by bilin chromphores covalently attached to the phytochrome protein [5], [68], [69], [70], [71] (Fig. 3h). The structure of phytochromes consists of an N-terminal photosensory core module and a C-terminal regulatory region, which includes a histidine-kinase-like domain. Phytochromes and related sensors produce a range of bilin derivatives such as phycocyanobilin (PCB), phytochromobilin (PΦB), biliverdin (BV) and phycoviolobilin (PVB) [72], [73]. These bilin chromophores are responsible for the light-absorption properties of phytochromes, allowing them to function as red/far-red light sensors.

### 2.10 Photocobilins

Contrary to the typical single photosensitive domain in natural photosensitive proteins, Zhang *et al*. discovered a new subclass of multi-centre photosensitive protein in nature, designated as Photocobilins (Photoactive-cobalamin-bilin, Pcob) [74]. This novel finding has revealed that Pcob possess two distinct photosensitive domains: CBD and the biliverdin-binding domain (BBD), which are co-localized within a single protein molecule [74]. By utilizing the cofactors B_12_ and BV, Pcob can simultaneously sense wavelengths across the entire visible light spectrum (Fig. 3j). It is noteworthy that a specific class of Pcob derived from *Acidimicrobiaceae bacterium* incorporates a diguanylate cyclase functional domain. This fusion of Pcob with an enzyme domain is representative of the complexity of these multi-centre photosensitive proteins and expands their potential roles in cellular metabolism and enzymatic regulation. The discovery of this novel dual-chromophore photosensitive protein provides a natural reference template for the future design of broad-spectrum optogenetic elements and photobiocatalysts.

### 2.11 Engineered photosensitive domains

In addition to the natural photosensitive domain, recent advances in engineered photosensitive domains have significantly expanded the spectral range and functional diversity of optogenetic tools through synergistic strategies combining directed evolution and rational design. Structural optimization of channelrhodopsins has yielded ChRmine, which achieves red-light responsiveness in deep tissues through a unique proton-coupled electron transfer mechanism [75], while deep mutational scanning of CreiLOV has overcome the oxygen-dependency limitations of traditional flavoproteins [76]. In modular photoswitch development, circularly permuted LOV2 has been reconfigured as programmable optical switches [77], and the LOV-Turbo system enables spatiotemporally precise proximity labeling in living cells via engineered allostery [78]. Notably, strategic mutations like Q63E in BLUF domains can lock photocycle intermediates in pseudo-activated states [79], while optimization of proton transfer pathways in AppABLUF significantly enhances blue-light responsiveness [80]. These engineered photoswitches not only surpass natural counterparts in sensitivity and specificity [81], but also enable red/far-red controllable gene therapies [82] and precise neural circuit analysis through axon-terminal optimized actuators [83]. The development of this multimodal, customizable optogenetic toolkit marks a new era in synthetic photobiology for precisely manipulating biomolecular interaction networks.

## 3 Optogenetic applications in cellular systems

Optogenetics has a wide range of applications in the cellular system, ranging from basic research to potential therapeutic strategies. This includes the precise control of neural circuits for neuroscience research, the regulation of gene transcription and protein localization for cellular signaling studies, and the development of light-controlled designer cells for synthetic biology and gene therapy. Optogenetics also shows great promise for stem cell research, optogenetic medicine, and the creation of light-responsive cellular systems for pharmaceutical production and drug screening, offering a precise and non-invasive approach for controlling biological processes with potential therapeutic and research implications.

### 3.1 From disease mechanism elucidation to translational therapies

**Fig. 4:**
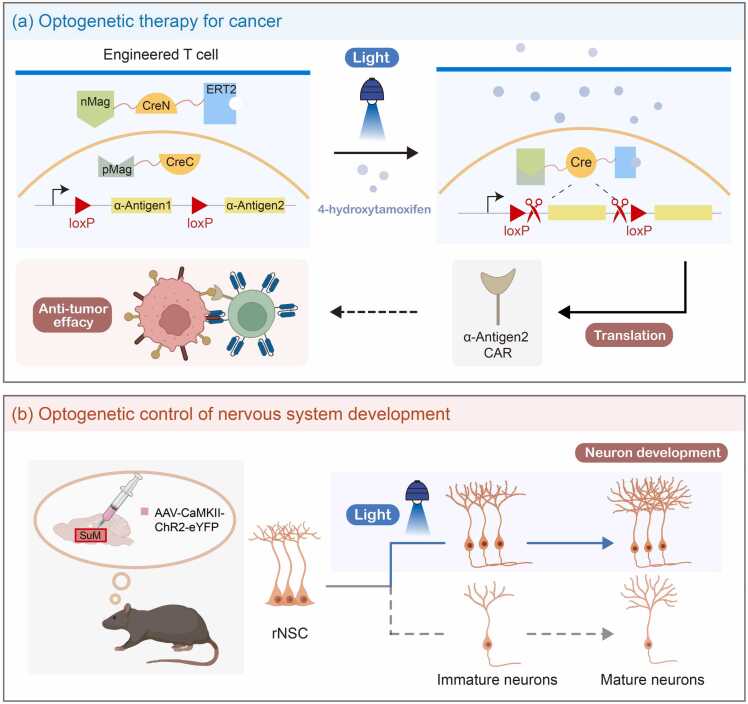
Applications of optogenentics. a. TamPA-Cre[95], The light-controlled heterodimer nMag and pMag were connected to the N-terminal and C-terminal of Cre recombinase, respectively. Under blue light irradiation, CreN and CreC were recombined into functional Cre, and the gene was recombined to express α-Antigen2, reducing the off-target rate of CAR-T therapy on cancer cells. b. AAV-CaMKII-ChR2-eYFP was injected into mouse SuM, and through continuous blue light stimulation, it was found that the activation of SuM could effectively promote the activation of rNSC and the process of rNSC to mature neurons[103]. Abbreviations: nMag, negative Magnet; pMag, positive Magnet; ERT2, estrogen receptor ligand binding domain; SuM, supramammillary nucleus neurons; rNSC, radial neural stem cells.

Optogenetics has emerged as a transformative tool in biomedical research, bridging the gap between basic neuroscience and therapeutic development. By utilizing photosensitive proteins, this technique enables precise exploration of disease mechanisms while promoting therapeutic strategies and streamlining drug discovery pipelines. In disease mechanism studies, optogenetics empowers researchers to decode complex neural circuits involved in conditions such as Parkinson's disease [84], epilepsy [85], [86], and depression [87], revealing previously inaccessible pathophysiology insights. In therapeutic applications, this approach demonstrates potential for enhancing cancer immunotherapy [88], [89], [90] and diabetes [91], [92], [93], [94] treatments, and improving CAR-T cell therapy [95], [96] (Fig. 4a). Furthermore, in drug development, optogenetic methods facilitate the real-time monitoring of drug effects on cellular signaling [97], [98], [99], [100], [101], thus accelerating the precision and speed of drug discovery [102]. Overall, optogenetics is redefining our approach to understanding and treating neurological and other diseases through its high temporal and spatial resolution capabilities.

**Fig. 5:**
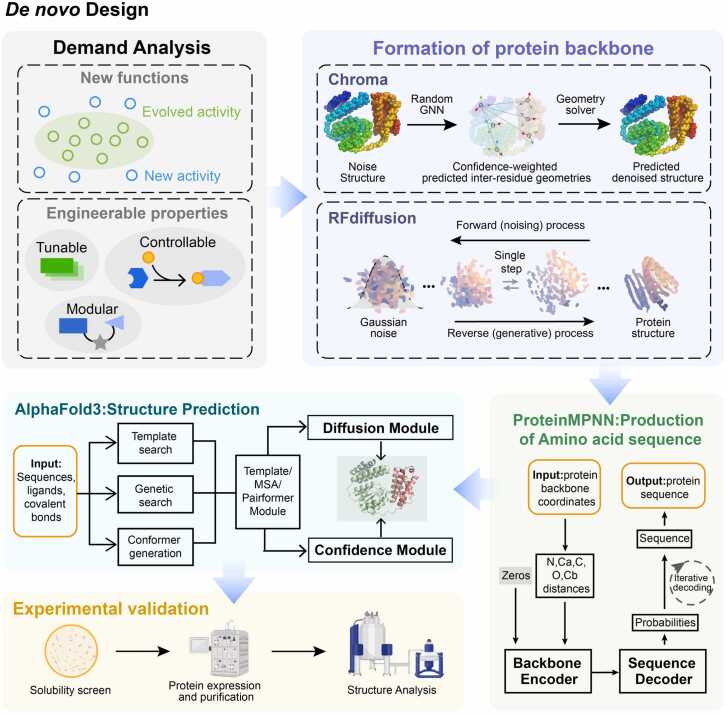
Schematic diagram of protein de novo design processes and AI-assisted tools.

### 3.2 Decoding neural circuit dynamics

Optogenetics has fundamentally transformed neuroscience by enabling the precise control of neuronal activity, which has profound implications for understanding brain function and behavior. Through the targeted expression of photoresponsive proteins, investigators can functionally map neuronal populations governing complex behaviors including arousal [104], feeding [105], [106], social interactions [107], [108], [109], [110], [111], [112] and influence of memory [113], by selectively activating or inhibiting targeted neural circuits. Klapoetke *et al.* demonstrated multiplexed control of distinct neural populations through wavelength-selective optogenetic actuators, enabling the simultaneous modulation of divergent circuits with minimal crosstalk [114]. Furthermore, advancements in optogenetics, including the development of integrated devices that can simultaneously stimulate and record [115], [116] and allow multaneously visualizing the resulting changes in brain activity, and the precise visualization of signal regulation [117], have enhanced the ability to study neural dynamics in real-time [103], [118], [119], [120], [121], [122], [123] (Fig. 4b). These innovations significantly enhance our comprehensive understanding of neural circuitry and also show potential for therapeutic applications in neuropsychiatric disorders and other neurological impairment conditions [85], [124], [125], [126].

Beyond mammalian systems, *Drosophila* has emerged as a powerful model for optogenetic studies. Light-gated tools such as the rapid induction of gene expression in fruit files [127] and optogenetic Gal4 systems [128] have enabled the precise interrogation of neural circuits and developmental processes, highlighting the cross-species applicability of optogenetic methodologies.

### 3.3 Spatiotemporal control of molecular networks and cellular signaling

Optogenetics has redefined molecular systems biology by enabling femtosecond-precision control over cellular processes. By employing optogenetics to achieve precise control over cellular processes, researchers manipulate signaling pathways, gene expression, and protein localization with high spatial and temporal resolution [129]. Notably, light-regulated nuclear import/export systems – such as those engineered by coupling LOV domains to nuclear localization signals or export signals – have enabled the dynamic control of transcription factor shuttling, allowing the continuous dissection of gene regulatory networks under subcellular confinement [130], [131].

For instance, optogenetic systems have been developed to control the activity of proteins involved in critical cellular functions, such as kinases, thereby elucidating their roles in various biological processes [132]. Additionally, optogenetic tools have been employed to investigate mechanotransduction—the process by which cells sense and respond to mechanical stimuli—by enabling the manipulation of cellular forces and signaling pathways in real-time [133]. Similarly, the development of novel optogenetic constructs, such as those based on microbial rhodopsins, has expanded the toolkit available for probing various cellular mechanisms, including apoptosis and calcium signaling [134], [135].

Multiplexed optogenetic control now supports the hierarchical manipulation of signaling pathways-simultaneously programming upstream receptor activation and downstream transcriptional responses within single-cell contexts [136]. Optogenetic tools have also been employed to dissect dynamic signaling pathways such as Notch signaling. For instance, studies on the desensitization of Notch signaling through nuclear dynamic adaption demonstrate how light-controlled perturbations can reveal temporal the regulation of transcriptional feedback loops [137].

Besides, optogenetic strategies are increasingly applied to regulate endogenous cellular components. For example, CRY2-based clustering systems have been engineered to modulate transmembrane receptor activity, offering a non-invasive approach to study receptor signaling and trafficking in situ [138]. Furthermore, cybergenetic frameworks integrating optogenetics with continuous computational feedback, such as cell-in-the-loop pattern formation and single-cell controllers, enable the dynamic interrogation of stochastic transcriptional regulation and synthetic ecosystem design [139], [140].

Collectively, optogenetic tools serve as a powerful platform for deconstructing cellular complexity, propelling mechanistic studies from static observations to dynamic system analyses.

The transformative impact of cellular optogenetics spans neuroscience, precision medicine, and molecular systems biology. Its capability to provide precise control over cellular activity has opened new avenues for research and therapeutic development, making it an invaluable tool in contemporary biological sciences. Future developments will likely focus on cell type-specific targeting, organelle-level precision, and closed-loop therapeutic systems, advancing in regenerative medicine and other fields.

## 4 Engineering photosensitive proteins for optogenetics

The field of optogenetics has revolutionized the approach researchers take to manipulate biological systems, enabling precise control over cellular processes using light. As the primary actuator of optogenetics, the design and optimization of photosensitive proteins lie at the core of the development of optogenetics. By employing novel methods of protein engineering, researchers create hybrid proteins that can activate or inhibit specific signaling pathways under light through inducing protein oligomerization, split-protein recombination, or fusing proteins with other functional domains. This modular strategy in protein design enables the creation of customized optogenetic tools that can be optimized for specific applications [141], [142].

The development of non-opsin-based photosensitive proteins has expanded the toolkit available for optogenetic applications. For example, LOV domains and phytochromes have been engineered to construct light-responsive systems that can regulate protein interactions [143], gene expression [144], and even metabolic pathways [145]. Similarly, some pioneering work in non-mammalian models driving these advances. In *Escherichia coli*, the fusion of LOV domains with histidine kinases enabled light-regulated two-component signaling, a strategy subsequently adapted for mammalian synthetic biology [146]. These engineered proteins offer distinct advantages, including the ability to operate in disparate spectral ranges, thereby facilitating multiplexing—the simultaneous modulation of multiple cellular pathways via different wavelengths of illumination [147]. Such projects also include **RinID**[148] and **cpLOV2**[149]. Engineered photosensitive proteins enable the precise control of gene expression and protein activity through light-induced conformational change and allow for the exploration of complex signaling pathways in living cells. For example, Motta-Mena *et al.* developed a blue-light-inducible gene expression system (pC120) with rapid activation and deactivation kinetics, achieving millisecond-scale control of transcriptional outputs [150]. These specific pathways have also been utilized in production activities, such as the interaction of CRY2 and Cib1 photosensitive domains, developed by Kennedy *et al.*[151], which has been employed to promote sustained high expression of sweet-protein in *Saccharomyces cerevisiae*[152].

**Table 1:** List of Optogenetic Tools (Based on Section 4).

Optogenetic Tools	domain	Wavelength	Optical response characteristic	Application	Reference
**Engineering Strategy**					
LOV-LexA	LOV	blue(450 nm)	Homodimerization	gene expression	46
PhotoGal4	phytochrome B	red(660 nm)	Heterodimerization	Tissue specific regulation of Drosophila melanogaster	47
OptoSTIM1	CRY2	blue(480 nm)	Homodimerization	Regulation of calcium signal in HeLa cells	190
LINuS	LOV2	blue(450 nm)	Conformational change	Dynamic regulation of transcription factors	131
optoACRs	LOV2	blue(450 nm)	Heterodimerization	Spatio-temporal specific gene editing	161
NIR-AC	BphG1	red(660 nm)	Heterodimerization	Deep tissue metabolic regulation	141
**AI-assisted Design**					
ChRmine	ChR	red(600 nm)	Homologous polymerization	Noninvasive transcranial neuron activation	75
REDMAP	phytochrome A	red(660 nm)	Heterodimerization	Deep tissue cAMP signal regulation	159
CreiLOV	LOV	blue(450 nm)	Conformational change	Hypoxic environmental optogenetics	76
SMOCs	LOV/ Phytochrome/ CRY2	blue(450 nm)/ red(600 nm)	Polymeric complex	Programmed innate immune regulation	153
cpLOV2	LOV2	blue(450 nm)	Conformational change	Dynamic regulation of kinase activity	77
**Therapeutic application transformation**					
REDLIP	DrBphP	red(660 nm)	Heterodimerization	Diabetes/Cancer gene therapy	160
ChRger2	ChR	red(660 nm)	Homologous polymerization	Noninvasive neuronal regulation	165
LiCAR-T	CRY2	blue(450 nm)	Heterodimerization	Precision immunotherapy for cancer	96
ChOp-FAK	LOV2	blue(450 nm)	Two-input logic gate	Study on cell migration and adhesion	185
LiSmore	CRY2	blue(470 nm)	Polymeric complex	Tumor immunotherapy	89

In addition to enhancing the performance of existing proteins, researchers are also exploring novel strategies for optogenetic control. One promising approach involves the use of synthetic protein condensates that can dynamically recruit and release proteins in response to light. This approach exploits the principles of phase separation and modular protein design to create systems that can rapidly respond to light stimulation, providing an additional level of control over cellular processes. Such designs are paving the way for more sophisticated optogenetic applications, particularly in mammalian systems where the complexity of signaling networks poses significant challenges [153].

Moreover, the rapid progress of engineered photosensitive proteins has significantly enhanced the precision and versatility of optogenetic tools in biological research. These applications, including the targeted degradation of proteins [154], [155], [156] and the manipulation of gene expression [157], are also expanding. Ye *et al.* developed REDMAP [158], a red-light-responsive system with high penetration, and REDLIP [159], a light switch that operates without the need for foreign chromophores in mammalian cells. Recent efforts in protein engineering have led to the development of novel photosensitive proteins with enhanced functionalities. For instance, Tan *et al.*'s engineering of supramolecular organizing centers **(SMOCs)** has facilitated programmable control over innate immune responses, demonstrating the potential for customized therapeutic applications [160]. Additionally, Hoffmann *et al.* presented a novel approach to regulate CRISPR-Cas9 activity through optogenetics by utilizing a specifically engineered anti-CRISPR protein that can be activated by light, which minimized off-target effects and enhanced the safety and efficacy of CRISPR applications in various biological contexts [161].

Beyond spectural tuning, novel optogenetic probes now incorporate environmental responsiveness. For instance, temperature-sensitive optogenetic systems enable dual control of signaling pathways through light and thermal stimuli, enhancing spatiotemporal precision in dynamic environments [162]. Similarly, tools like BcLOVclust achieve rapid, light-induced cytoplasmic protein clustering, enabling real-time manipulation of intracellular processes such as phase separation and signal amplification [163]. These innovations exemplify the growing versatility of optogenetic actuators in probing complex cellular dynamics.

The integration of machine learning and computational approaches in the design of optogenetic tools has accelerated the optimization process. By predicting how modifications to protein structure will impact function, researchers can efficiently streamline the engineering process, thereby reducing the time and resources required to develop effective optogenetic actuators [164], [165]. The computational approach synergistically complements traditional experimental methods, offering a more comprehensive strategy for designing proteins with desired characteristics.

Recent advances extend optogenetics to mechanical regulation within tissues. For instance, light-controlled modulation of cell contractility during morphogenesis [166] showcases how engineered proteins can interact with cytoskeletal dynamics, offering new insights into biomechanical signaling in development and disease.

In summary, modifying or creating photosensitive proteins through protein engineering is the cornerstone of optogenetics, facilitating the development of sophisticated tools for the photocontrol of biological processes. The ability to customize these proteins for specific uses enhances their utility in fundamental research and clinical application. The integration of new engineering strategies and computational protein design will continue to drive the further development of optogenetic technologies.

## 5 Computational protein design accelerating optogenetics tools development

### 5.1 Current status of computational protein design

Protein design enables both modification of existing proteins and creation of novel protein architectures, with broad applications across biotechnology. In the early stages of protein design during the 1980s, the prevalent approach relied on rational and minimal design principles, where residue positioning was induced based on physicochemical and biological characteristics, with limited computational assistance. However, advancements in algorithms, improvements in hardware technology, and the growing demands for customization have solidified the significance of computational protein design in contemporary research and applications [167]. Nowadays, protein design employs two primary strategies: template-based design and *de novo* design. Template-based design exploits the existing functional and structural features of proteins, thereby enhancing pre-existing protein scaffolds with new functionalities. In contrast, *de novo* design involves reconstructing the protein backbone and sequence from the very beginning based on specific design criteria, while adhering to the principles of physical structure and chemistry [168], [169].

Recently, the field of protein design has undergone a remarkable transformation with the advent of artificial intelligence (AI) tools, transitioning from traditional biochemical methodologies to data-driven sophisticated algorithms. The latest Nobel Prize in Chemistry has underscored the significant impact of AI in the field of protein design. The 2024 Nobel Prize in Chemistry was awarded to three scientists for their groundbreaking work on proteins. David Baker from the University of Washington received half of the prize for his contributions to "computational protein design", while the other half was jointly awarded to Demis Hassabis and John M. Jumper from Google DeepMind for their work on "protein structure prediction". Demis Hassabis and John M. Jumper developed an AI model (**AlphaFold)**[29], [170] that fundamentally changed the way we study protein structures. AlphaFold is capable of accurately predicting the 3D structure of proteins from their amino acid sequences, a problem that had been unsolved for 50 years. The groundbreaking work of AlphaFold has also catalyzed the development of other AI models for protein structure prediction by setting a new benchmark for accuracy and efficiency, such as **RoseTTAFold**[171] and **OpenFold**[172]. The latest version of **AlphaFold3**[173] employs a diffusion-based model architecture that efficiently predicts conformations of complexes, including proteins, nucleic acids, small molecules, ions, and modified residues, facilitating high-precision modeling across diverse biomolecular spaces within a unified deep learning framework, setting new standards for accuracy.

The advent of protein structure prediction has revolutionized protein design by enabling the creation of novel proteins with tailored functions, significantly enhancing the precision of protein structures prediction from amino acid sequences, and providing invaluable guidance for complex protein engineering. This technological leap has also had a profoundly impact on protein design by expanding the **Sequence-Structure-Function** space, substantially increasing the speed and accuracy of design and optimization, and considerably facilitating high-throughput screening to identify candidates with the desired characteristics. Additionally, tools like **ProALIGN**[174], which specialize in identifying conserved regions, significantly contribute to the study of evolutionary and functional similarities. **MUSE**[175] introduces a multi-scale learning framework that integrates atomic and molecular information, demonstrating exceptional performance in predicting intermolecular interactions. The new methods promote the design of specific functional proteins, allowing researchers to analyze the structural function of proteins from multiple aspects of evolution, sequence, and structure, while predicting structural and functional changes in protein mutants.

AI tools have significantly enhanced structural prediction accuracy and also expanded the scope of protein structure backbone generation and sequence design. For example, **RFdiffusion**[30], built upon RoseTTAFold, has developed a protein backbone generation model, which demonstrating high efficiency and accuracy across various structural designs. **ProteinMPNN**[176] utilizes graph neural networks for protein sequence design, efficiently capturing and leveraging structural information to generate protein backbones with robust stability and functionality, achieving high success rates in experimental validations and computational efficiency.

Furthermore, AI-driven methodologies have enabled the customization of protein functions tailored to specific applications in medicine, biotechnology, and synthetic biology. Lutz *et al.*[177] integrated reinforcement learning with top-down protein design, optimizing amino acid sequences progressively from a structural and functional perspective, thereby enhancing folding stability and functionality. By analyzing a large amount of protein sequence and structural data, **Chroma**[178] predicts folding modes for directional functional variant generation, so that protein variants with specific functions or properties can be quickly generated in a targeted manner, significantly improving the efficiency and success rate of protein design. Combined with RFdiffusion for the generation of functional structures, and ProteinMPNN and Chroma for the generation of high-quality sequences with stable folding, high expression, solubility and other features, the researchers were able to solve complex functional protein design challenges that were previously difficult to address using traditional methods. And the superiority of the algorithm greatly improves the efficiency of the workflow. The synergistic and complementary relationship between these tools enhances the overall efficacy of computational protein design, enabling the exploration of complex biological systems and the development of novel therapeutic strategies.

In conclusion, the extensive application of AI tools in protein design marks a significant milestone in the development of proteins with customized structures and functions. Despite ongoing challenges in establishing conformational dynamics models and generating novel proteins that function stably in physiological environments, the continuous evolution of computational methods is expected to enhance the potential for designing new proteins customized for applications in medicine, biotechnology, and synthetic biology. This progress opens up new possibilities to delivering cutting-edge strategies for further development across multiple disciplines [170].

### 5.2 AI-driven computational design of mammalian-optimized for precision optogenetics and therapy

Computational protein design has emerged as a pivotal approach in the development of proteins tools for mammalian cells. While the principles of computational protein design are universal, mammalian systems pose unique challenges due to their complex secretory machinery, dense signaling networks, and therapeutic translation requirements. Recent breakthroughs in AI-driven protein design methodologies now enable the systematic optimization of optogenetic actuators for precise neuromodulation.

The integration of computational design techniques allows for the systematic exploration of protein sequence-structure relationships. For instance, methods such as dead-end elimination (DEE), guarantee the design of proteins that not only fold correctly but also exhibit desired functionalities [179]. Havranek *et al.* also highlight the importance of specificity in computational protein design, which is crucial for creating proteins that can interact with their targets with high affinity and selectivity [180]. This specificity is particularly vital in therapeutic applications, where the designed proteins must interact precisely with biological targets to elicit the desired therapeutic effects.

Moreover, protein design facilitates the development of novel protein-based systems with potential therapeutic applications. For example, the design of supercharged proteins for macromolecule delivery into mammalian cells has shown substantial efficacy in enhancing the effectiveness of drug delivery systems [181]. This approach leverages computational insights to redesign proteins that can effectively penetrate cellular membranes and deliver therapeutic agents. Bojar and Fussenegger also emphasize the role of protein design in developing novel biomedical applications. Redesigned proteins can be integrated into gene circuits to create sophisticated cellular behaviors, which can be harnessed for therapeutic purposes [182]. Pistikou *et al.* propose an orthogonal platform for synthetic communication in mammalian cells, potentially enabling the design of therapeutic systems that mimic the human immune response [183]. Bedbrook *et al.* employed machine learning models to engineer multiple ChRs variants. By optimizing the photosensitivity and photocurrent amplitude of these ChRs, their approach significantly reduced off-target effects in optogenetic interventions. Notably, the variant ChRger2 achieved transcranial light-induced neuronal activation without requiring fiber impantation, marking a critical advancement in minimally invasive neuromodulation [165]. Such systems could revolutionize cell-based therapies by allowing for more precise control over cellular functions.

Furthermore, protein design can provide novel tools for modulating cellular activity and behavior. Pillai *et al.* explores the design of allosterically switchable protein assemblies through the rigid-body coupling of effector-switchable hinge modules [184]. This approach allows for the generation of diverse allosterically switchable systems, including cyclic rings that can incorporate or eject subunits in response to effector binding. In addition, Vishweshwaraiah *et al.* used computational methods to identify sites and optimized the protein structure through molecular dynamics simulations, embedding two regulatory domains containing LOV2 into the kinase focal adhesion kinase(FAK). This allowed for dynamic regulation of FAK activity using chemical and optogenetic switches, functioning as a “two-input logic OR gate” [185]. Building on thses advancements, a recent study by Ong et al. demonstrated the optogenetic control of Protein Kinase Cepsilon (PKCε) activity, revealing its intrinsic signaling properties with spatiotemporal resolution [186]. By integrating light-sensitive domains into PKCε, the researcheres were able to precisely control its activation and deactivation in live cells, providing new insights into the role of PKCε in cellular signaling pathways. These studies demonstrates the potential of protein design for applications in synthetic biology and therapeutic interventions.

In conclusion, the integration of AI-assisted protein design with mammalian cell engineering has emerged as a cornerstone of modern optogenetic innovation. By leveraging computational tools such as AlphaFold for structure-guided optimization and RFdiffusion for *de novo* generation of photoactuators, researchers now systematically address long-standing challenges in optogenetics – from enhancing tissue-penetrating excitation wavelengths to achieving cell-type-specific neuromodulation with minimal off-target effects.

### 5.3 Advancements in optogenetic protein design: enhancing specificity and functionality

Initially, the photosensitive proteins upon which researcheres developed optogenetic tools were predominantly derived from microorganisms and plants. However, the inherent photoreactive properties and limited operational mechanisms of these natural proteins often fail to meet the diverse requirements across various applications. Consequently, protein design plays a pivotal role in surmounting these limitations, enabling the development of novel therapeutic approaches and expanding the scope of its applicability.

Protein design significantly enhances the specificity and targeted applicability of photosensitive proteins. Through targeted modifications to natural photoreceptive domains, such as LOV and CRY2, these domains have been redesigned to possess distinct photoreceptive characteristics and regulatory functions, ultimately resulting in enhanced specificity and targeted applicability [149], [151], [187], [188]. For instance, the engineering of photoactivatable adenylate cyclases in Euglena gracilis enabled blue-light-controlled cAMP production, a concept subsequently translated into mammalian systems for the precise modulation of GPCR signaling [189]. In the OptoSTIM1 system, the C-terminal fusion of a short peptide to CRY2PHR significantly increased protein aggregation under blue-light illumination, allowing for precise control over calcium signaling in HeLa cells and doubling the process [190]. Continuing to advance the field, a light- and calcium-gated transcription factor has been developed for imaging and manipulating activated neurons, providing a powerful tool for studying neuronal activity with high spatiotemporal precision [191], which highlights the potential of integrating photosensitive protein engineering with calcium signaling.

Emerging platforms now combine optogenetics with single-cell resolution to dissect stochastic gene regulation. A recent study demonstrated real-time, light-controlled interrogation of transcriptional bursting in individual cells, revealing how dynamic optogenetic perturbations can unravel noise-driven regulatory mechanisms [192]. Such approaches highlight the synergy between computational design, high-resolution optogenetic tools, and single-cell analytics in advancing synthetic biology.

Furthermore, the design of novel proteins has enabled innovative optogenetic applications, facilitating continuous monitoring or manipulation of cellular physiological processes. Gil *et al*. developed OptoNBs, a novel, multifunctional chimeric switch protein specifically designed to control target binding and modulate signaling pathways in response to light exposure. This capability enables precise manipulation of cellular process both *in vivo* and *in vitro*[193]. Similarly, structural modifications enable alterations to protein trafficking pathways in targeted cells by redesigning key binding sites and interactions that govern cellular uptake. Lerner *et al*. embedded nuclear localization/export signal sequences within the LOV2 photoreceptive domain, achieving light-responsive transport of the target protein [194]. Similar work includes the light-induced nuclear localization signal LINus developed by Niopek et al. [131]. This advancement is valuable for studies exploring the spatial dynamics of cellular motility and variations in signaling pathway.

In summary, integrating computational protein design into the development of optogenetic has significantly enhanced our capacity to manipulate mammalian cell functions with unparalleled precision and specificity. As protein design technologies continue to progress, we can anticipate the emergence of next-generation optogenetic tools that offer a high degree of design flexibility, further expanding the utility and efficacy of optogenetic interventions in various biological and medical applications.

### 5.4 Current challenges in optogenetic development

**Fig. 6:**
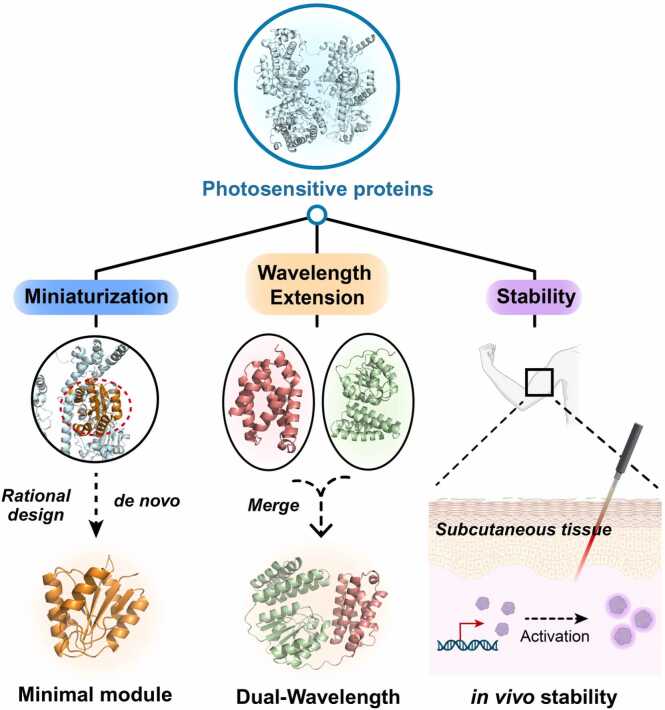
Three primary strategic directions for optogenetic elements to address development challenges. The schematic diagram shows the design ideas and application scenarios of the three development strategies of optogenetic elements.

While optogenetics has revolutionized biological manipulation, substantial challenges still remain in further advancing its applications. Below, we systematically outline the key limitations and propose strategic solutions (Fig. 6).

#### 5.4.1 Miniaturization and modular integration

The relatively large size of some optogenetic proteins restricts their modular integration into protein complexes. Developing smaller and more efficient photosensitive proteins can enhance precision by identifying and simplifying the non-active regions of proteins while retaining essential functional domains [149]. This approach has the potential to enhance the modularity of photosensitive components, increasing their potential for integration into protein complexes or regulatory systems. A high-throughput optogenetic platform can accelerate the development and optimization of optogenetic tools. For instance, Lustro, a automated system enables large-scale screening of optogenetic constructs in yeast, facilitating rapid characterization of light-responsive behaviors and multiplexed control of cellular pathways [195], [196]. Such platforms bridge the gap between computational design and experimental validation, offering a scalable framework for refining optogenetic elements across diverse biological contexts. Another strategies is AI-guided downsizing based on RFdiffusion backbone generation and ProteinMPNN sequence design.

#### 5.4.2 Spectral and functional diversity

Current optogenetic tools primarily operate within the blue-green wavelength range, constraining multiplexed control and deep-tissue applications. Protein design can be employed to fuse photosensitive domains with enzymatic functional domains, transforming target enzymes into stable light-activated enzymes that enable pathway regulation. A pioneering example of this approach is provided by Ryu *et al.*, who engineered a near-infrared(NIR) light-regulated adenylate cyclase by fusing a bacterial phytochrome with the catalytic domain of adenylate cyclase(AC) from *Beggiatoa* sp. [141]. This chimeric protein, termed NIR-AC, utilizes NIR light(740 nm) to induce cyclic AMP(cAMP) production, enabling precise spatiotemporal control of cAMP signaling pathways in mammalian cells. Additionally, introducing two photosensitive domains within the same protein may expand the range of responsive wavelengths, enabling multifunctional regulation across different wavelengths. Zhang S. *et al.* recently discovered Pcob protein, which serves as a reference for such research [74]. Similarly, prior to Zhang’s elucidation and validation of the structure and function of the Pcob protein, researcheres had already predicted a novel photosensitive protein with a dual photoreceptive domain through bioinformatics analysis. This underscores the significant role of data analysis in the discovery of new photosensitive proteins [197].

#### 5.4.3 Functional stability in physiological environments

In the field of medicine, light transmission at certain intensities through human tissues entails varying degrees of attenuation, imposing stringent requirements on responsive wavelengths and the sensitivity of photosensitive component. Expanding the photosensitive domain of photosensitive proteins from visible light to infrared addresses the issue of limited blue-light penetration, enabling intracellular photosensitive elements to respond to low-energy light sources and allowing for deeper tissue penetration [198]. Moreover, newly designed proteins must endure physiological conditions and maintain stable expression within the body, necessitating computational simulations of protein design coupled with thorough validation using animal models. This approach is critical for enhancing the translational efficiency of optogenetic tools across diverse biomedical environments.

#### 5.4.4 Spatiotemporal precision and off-target effects

Despite the high theoretical precision of optogenetics, practical limitations arise from the crosstalk caused by the overlapping spectral sensitivities of multi-component systems and basal activity, for example, the dark-state leakage of photoswitches [79]. The progress made thus far provides us a direction, including logic-gated systems like the "two-input OR gate" FAK kinase requiring simultaneous light/chemical activation [185], and AI-optimized tools such as ChRger2, where machine learning reduced off-target neuronal activation [165].

#### 5.4.5 Translational barriers in clinical applications

Clinical adoption faces hurdles like invasive light-delivery requirements and regulatory complexities for gene-circuit control (e.g., CAR-T safety validation) [96]. Emerging solutions include battery-free wireless implants for programmable light delivery [21], [94] and "cell-in-the-loop" platforms integrating computational feedback [139]. However, scaling these technologies for long-term human use necessitates rigorous biocompatibility testing and standardization of dosing protocols to strike a balance efficacy and safety.

## 6 Summary

Optogenetics has revolutionized the manipulation of biological processes in cellular systems, with protein design driving the creation of tailored tools. By integrating computational methods (e.g., AlphaFold, RFdiffusion) and AI-driven optimization, researchers now systematically tackle challenges such as spectral tuning and cell-type specificity. While substantial progress has been achieved, ongoing efforts are focused on enhancing tool miniaturization, multiplexed regulation, and therapeutic translation. These advancements will broaden the utility of optogenetic in both fundamental research and clinical applications.

## Author statements

We the undersigned declare that this manuscript is original, has not been published before and is not currently being considered for publication elsewhere. We confirm that the manuscript has been read and approved by all named authors and that there are no other persons who satisfied the criteria for authorship but are not listed. We further confirm that the order of authors listed in the manuscript has been approved by all of us. We understand that the Corresponding Author is the sole contact for the Editorial process and is responsible for communicating with the other authors about progress, submissions of revisions and final approval of proofs.

## CRediT authorship contribution statement

**Wang Yuxuan:** Writing – review & editing, Writing – original draft. **Zhang Shaowei:** Writing – review & editing, Supervision, Funding acquisition, Conceptualization. **Zhang Guohao:** Writing – review & editing, Investigation. **Wu Guohua:** Writing – review & editing, Visualization, Investigation. **Wu Xiaomin:** Writing – review & editing, Visualization, Project administration, Investigation. **Zhu Lingyun:** Writing – review & editing, Writing – original draft, Supervision, Investigation, Funding acquisition. **Liu Chuanyang:** Writing – review & editing, Writing – original draft, Investigation, Conceptualization.

## Declaration of Competing Interest

The authors declare no competing interests. Correspondence and request for materials should be addressed to C.L. and S.Z.
